# Predicting survival of Hodgkin lymphoma using machine learning-an analysis based on the SEER database

**DOI:** 10.1007/s00277-026-06791-x

**Published:** 2026-04-30

**Authors:** Xinzhen Cai, Lili Kang

**Affiliations:** Department of Hematology, Gaoyou People’s Hospital, Jiangsu Province China, 225600 China

**Keywords:** Hodgkin lymphoma, Machine learning, Survival analysis, SEER database, Population-based study

## Abstract

**Objectives:**

This study aimed to develop an effective model for predicting Hodgkin lymphoma (HL) prognosis as to assist clinicians in making optimal clinical decisions.

**Methods:**

This study screened HL patients from the Surveillance, Epidemiology, and End Results (SEER) database from 2000 to 2021. Feature selection was performed using the Boruta algorithm. Four ML models were built based on the feature selection algorithm. The area under the curve (AUC), decision curve analysis, and Brier score were employed to evaluate the reliability of the four ML models. The feature importance was ranked through the Shapley Additive Explanation (SHAP). Based on the results of the SHAP plot, Kaplan–Meier analysis was used to compare the survival probabilities among different groups.

**Results:**

Among the 11,259 enrolled HL patients, 8,928 were alive and 2,331 had died. Primary site, year of diagnosis, B symptoms, surgery, marital status, Ann Arbor stage, radiation, SEER stage, chemotherapy, delay (diagnosis to treatment), age were associated with HL overall survival (OS). Of four ML models, the eXtreme Gradient Boosting (XGBoost) model exhibited superior predictive performance. For predicting 1-year OS, the net benefit of XGBoost, Cox proportional hazards (Coxph), and Random Survival Forest (RSF) models was significantly higher than that of the Light Gradient Boosting Machine (LightGBM) model, the treat-all model, and the treat-none model. Age, Ann Arbor stage, B symptoms, marital status, and radiation were the top five indicators in the feature importance ranking for HL OS.

**Conclusion:**

The XGBoost had excellent predictive performance in the prognostic model, which further helps clinicians to select appropriate treatment options.

**Trial registration:**

Not applicable.

**Supplementary Information:**

The online version contains supplementary material available at 10.1007/s00277-026-06791-x.

## Introduction

Hodgkin lymphoma (HL) is characterized by the presence of abnormal Reed-Sternberg (R-S) cells in lymph nodes [1]. It primarily affects young adults (20–40 years) and the elderly, with a slight male predominance, accounting for 10%–15% of all lymphomas [2]. Globally, there were 82,409 new HL cases and 22,701 HL-related deaths in 2022 [3]. While advancements in treatments, including Adriamycin, Bleomycin, Vinblastine, Dacarbazine (ABVD) chemotherapy, immune checkpoint inhibitors, and targeted therapies, have significantly improved patient survival [4–6], challenges remain: long-term treatment toxicity, particularly in early-stage patients [7], and poor outcomes in refractory or relapsed disease [8]. Therefore, developing an accurate prognostic model is imperative to predict survival outcomes, reduce overtreatment in low-risk patients, identify high-risk populations early, and implement more aggressive treatment measures.

The prognosis of HL patients is associated with multiple factors, such as age, stage, and B symptoms [9–11]. The commonly used scoring systems for HL include the Ann Arbor staging system (Ann Arbor stage) and 7-factor International Prognostic Score (IPS-7) [12, 13]. The latter is applicable for predicting the long-term survival of patients with stage III-IV Hodgkin lymphoma. The IPS-7 includes parameters such as age, performance status, and laboratory findings, but lacks relevant histopathological parameters, limiting its predictive accuracy. Given the limitations of existing models, there is a need for a more comprehensive and accurate scoring system.

Machine learning (ML) holds great potential in medical prognosis, as it can handle complex non-linear relationships and high-dimensional data [14, 15]. The U.S. Surveillance, Epidemiology, and End Results (SEER) database, which provides extensive long-term follow-up data on HL patients, offers an ideal platform for training and validating ML models. This study aims to utilize SEER data to systematically evaluate the performance of multiple ML algorithms in predicting HL prognosis, thereby facilitating the identification of high-risk HL patients in clinical practice and enabling the delivery of active and effective treatments.

## Materials and methods

### Study population

**Fig. 1:**
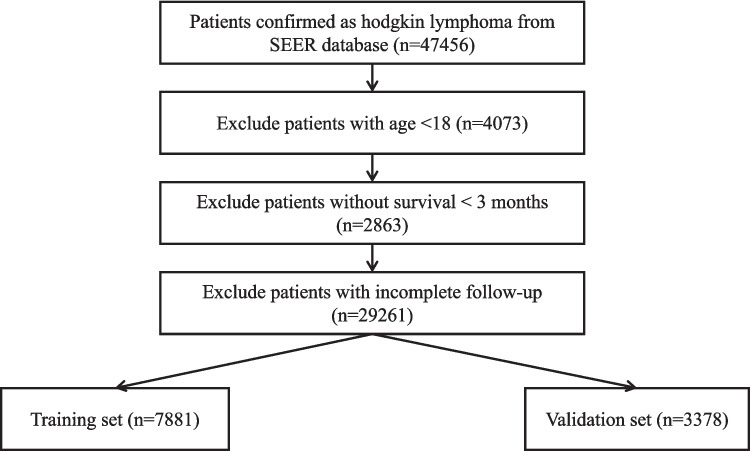
Flow chart of patient screening

The data used in this study were retrieved from the SEER database. The SEER database, an authoritative source of information on cancer incidence and survival rates in the United States, provides substantial data support for cancer research and epidemiological investigations, enabling analyses of cancer incidence trends, population-specific disease characteristics, treatment outcome evaluations, and prognosis predictions. In this study, SEER*Stat software was used to identify patients diagnosed with HL from the SEER database between 2000 and 2021. In this study, a total of 47,456 HL patients were initially retrieved from the SEER database. After excluding 4,073 patients aged < 18 years, 2,863 patients with a survival time of < 3 months, and 29,261 lost-to-follow-up patients, a final cohort of 11,259 patients was included for analysis. The patient selection process was illustrated in Fig. 1. In this study, ethical approval and informed consent were waived for this study because the data were obtained from the SEER database, a publicly accessible repository.

### Data collection

The primary endpoints of this study were the 1-, 3- and 5-year overall survival (OS) of HL patients. OS refers to the time from the diagnosis to death due to any cause. The following variables were extracted from the SEER database: gender (female, male), age (≤ 44,45–64, ≥ 65), race [Asian and Pacific Islander/American Indian/Alaska Native (AP/AI/AN), black, white, unknown], marital status (single, married, unknown), primary site [single region involved, multiple regions, lymph node, not otherwise specified (NOS)], chemotherapy (no, yes), radiation (no, yes), surgery (no, yes), year of diagnosis (2010–2014, ≥ 2015), ethnicity (non-Spanish-Hispanic-Latino, Spanish-Hispanic-Latino), median income (< $50,000, $50,000-$70,000, > $70,000), residential area (metropolitan, non-metropolitan), delay ( 0–1 month,1–2 months, 2–3 months, > 3 months, unknown), B symptom (none, any, unknown), Ann Arbor stage (I, II, III, IV, unknown), SEER stage (distant, localized, regional, unknown), survival months.

Age, gender, race, ethnicity, marital status, median income, radiation, primary site, year of diagnosis, chemotherapy, Ann Arbor stage, SEER stage, B symptoms, and surgery have all been identified to be associated with the survival of HL patients [6, 16–22]. Residential area, delay, survival monthswere included based on data availability in the SEER database.

### Feature selection and validation strategy

To minimize the risk of overfitting, feature selection was performed to remove irrelevant or redundant features. In this study, the Boruta algorithm was adopted to systematically evaluate the importance of features. The Boruta algorithm achieves all-relevant feature selection through statistical tests based on shadow features. It effectively handles high-dimensional data and data with complex interactions, is automated and highly robust, and can improve model performance [23–25]. Variables with *P* < 0.05 were ultimately included in the prognostic models. Additionally, the importance of each feature in prognostic models was ranked through the Shapley Additive Explanation (SHAP).

The entire dataset collected from the SEER database was randomly divided into a training cohort and a validation cohort at a ratio of 7:3. In the prognostic model, the area under the curve (AUC), decision curve analysis (DCA), Brier score was applied to assess the reliability of the four ML models. After a comprehensive comparison of different ML models, the model with the best predictive performance was selected as the final prognostic model. To further confirm the applicability of the selected model, we evaluated it in the validation cohort.

### ML algorithms

R software (version 4.4.3) software was used to construct the ML prediction models.

The Light Gradient Boosting Machine (LightGBM) is a tree-based ensemble learning method that employs gradient boosting technology. It combines multiple weak learners into a powerful model and is used to solve classification and regression problems. The LightGBM model was selected due to its efficiency in handling high-dimensional clinical data and its ability to capture non-linear relationships [26–28].

The Extreme Gradient Boosting (XGBoost) is a composite algorithm formed by combining base functions and weights, which exhibits excellent data fitting performance. Unlike traditional gradient boosting decision trees, XGBoost adds a regularization term to the loss function. Since some loss functions are difficult to differentiate, XGBoost uses the second-order Taylor expansion of the loss function for fitting. The XGBoost model was chosen because of its excellent performance in survival prediction tasks; it has been widely applied in the prognosis of cancer patients [29] and, in particular, shows significant advantages when dealing with imbalanced clinical data [30].

The Cox Proportional Hazards (Coxph) is used to analyze the impact of covariates on survival time in survival data. Based on a semi-parametric method, this model does not require specific assumptions about the hazard function. Instead, it uses the hazard ratio (HR) to compare the risks among different covariate groups. The Coxph model was selected as it is a classic survival analysis method [31] and can directly quantify the impact of covariates on risk in scenarios involving censored data [32, 33].

Random Survival Forest (RSF) is a survival analysis method based on the random forest. It constructs a large number of survival trees and determines the final prediction result through voting or weighting from individual trees. The RSF model was selected due to its robustness to censored survival data and its ability to model complex interaction effects without parametric assumptions [34].

### Statistical analysis

R 4.4.3 software was used for data description and statistical analysis. Normally distributed quantitative data were presented as mean [standard deviation (SD)] and analyzed using the Student’s t- test. Non-normal quantitative distributed data were presented as median (interquartile range [IQR]) and analyzed using the Mann–Whitney U test. Categorical variables were described as number (percentages) and analyzed using the chi-square test. The Boruta algorithm was used to screen important features. Variables with *P* < 0.05 were included in the four ML models (LightGBM, XGBoost, Coxph and RSF). Based on the results of the SHAP plot, Kaplan–Meier (KM) analysis was used to compare the survival probabilities among different groups. A difference was considered statistically significant when the *P* < 0.05.

## Results

### Patient demographics

Among the 11,259 enrolled HL patients, 8,928 were alive and 2,331 had died. In the HL population, HL primarily occurred in young adults aged ≤ 44 years, accounting for 55.7% of the total cohort. The majority of patients were white (80.7%) and male (55.2%). Except for ethnicity, statistically significant differences were observed in all variables between alive and dead patients, including gender, age, race, marital status, primary site, chemotherapy, radiation, surgery, diagnosis year, median income, residential area, delay (diagnosis to treatment), B symptoms, Ann Arbor stage, SEER stage, and survival months (Table 1).

**Table 1:** Baseline Characteristics of Patients

	level	Overall	Alive	Dead	*P*
n		(N = 11,259)	(N = 8928)	(N = 2331)	
Gender, n (%)	Female	5042 (44.8)	4103 (46.0)	939 (40.3)	< 0.001
	Male	6217 (55.2)	4825 (54.0)	1392 (59.7)	
Age, n (%)	≤ 44	6266 (55.7)	5828 (65.3)	438 (18.8)	< 0.001
	45–64	3026 (26.9)	2355 (26.4)	671 (28.8)	
	≥ 65	1967 (17.5)	745 (8.3)	1222 (52.4)	
Race, n (%)	AP/AI/AN	688 (6.1)	566 (6.3)	122 (5.2)	< 0.001
	Black	1345 (11.9)	1057 (11.8)	288 (12.4)	
	White	9090 (80.7)	7176 (80.4)	1914 (82.1)	
	Unknown	136 (1.2)	129 (1.4)	7 (0.3)	
Primary site n (%)	Single region involved	3497 (31.1)	2785 (31.2)	712 (30.5)	< 0.001
	Multiple regions	6181 (54.9)	5010 (56.1)	1171 (50.2)	
	Lymph node, NOS	1581 (14.0)	1133 (12.7)	448 (19.2)	
Marital status n (%)	Single	5476 (48.6)	4442 (49.8)	1034 (44.4)	< 0.001
	Married	5123 (45.5)	3975 (44.5)	1148 (49.2)	
	Unknown	660 ( 5.9)	511 ( 5.7)	149 ( 6.4)	
Chemotherapy, n (%)	No	1673 (14.9)	1198 (13.4)	475 (20.4)	< 0.001
	Yes	9586 (85.1)	7730 (86.6)	1856 (79.6)	
Radiation, n (%)	No	8259 (73.4)	6297 (70.5)	1962 (84.2)	< 0.001
	Yes	3000 (26.6)	2631 (29.5)	369 (15.8)	
Surgery, n (%)	No	8584 (76.2)	6745 (75.5)	1839 (78.9)	0.001
	Yes	2675 (23.8)	2183 (24.5)	492 (21.1)	
Year of diagnosis, n (%)	2010–2014	9387 (83.4)	7372 (82.6)	2015 (86.4)	< 0.001
	≥ 2015	1872 (16.6)	1556 (17.4)	316 (13.6)	
Ethnicity, n (%)	Non-Spanish-Hispanic-Latino	9339 (82.9)	7385 (82.7)	1954 (83.8)	0.216
	Spanish-Hispanic-Latino	1920 (17.1)	1543 (17.3)	377 (16.2)	
Median income, n (%)	< $50,000	790 (7.0)	582 (6.5)	208 (8.9)	< 0.001
	$50,000-$70,000	3474 (30.9)	2737 (30.7)	737 (31.6)	
	> $70,000	6995 (62.1)	5609 (62.8)	1386 (59.5)	
Residential area, n (%)	Metropolitan	10,101 (89.7)	8050 (90.2)	2051 (88.0)	0.002
	Non-metropolitan	1158 (10.3)	878 (9.8)	280 (12.0)	
Delay (diagnosis to treatment), n (%)	0–1 month	6331 (56.2)	5191 (58.1)	1140 (48.9)	< 0.001
	1–2 months	2376 (21.1)	1924 (21.6)	452 (19.4)	
	2–3 months	589 (5.2)	427 (4.8)	162 (6.9)	
	> 3 months	433 (3.8)	315 (3.5)	118 (5.1)	
	Unknown	1530 (13.6)	1071 (12.0)	459 (19.7)	
B symptom, n (%)	Any	4463 (39.6)	3445 (38.6)	1018 (43.7)	< 0.001
	None	5417 (48.1)	4483 (50.2)	934 (40.1)	
	Unknown	1379 (12.2)	1000 (11.2)	379 (16.3)	
Ann Arbor stage, n (%)	I	1762 (15.6)	1401 (15.7)	361 (15.5)	< 0.001
	II	4357 (38.7)	3824 (42.8)	533 (22.9)	
	III	2442 (21.7)	1808 (20.3)	634 (27.2)	
	IV	2146 (19.1)	1486 (16.6)	660 (28.3)	
	Unknown	552 (4.9)	409 (4.6)	143 (6.1)	
SEER stage, n (%)	Distant	4588 (40.7)	3294 (36.9)	1294 (55.5)	< 0.001
	Localized	1762 (15.6)	1401 (15.7)	361 (15.5)	
	Regional	4357 (38.7)	3824 (42.8)	533 (22.9)	
	Unknown	552 (4.9)	409 (4.6)	143 (6.1)	
Survival months, mean (SD)		89.34 (38.3)	102.50 (26.6)	38.93 (34.5)	< 0.001

SEER: Surveillance, Epidemiology, and End Results, AP/AI/AN: Asian and Pacific Islander/American Indian/Alaska Native; NOS, not otherwise specified

### Influencing factors for HL OS

**Fig. 2:**
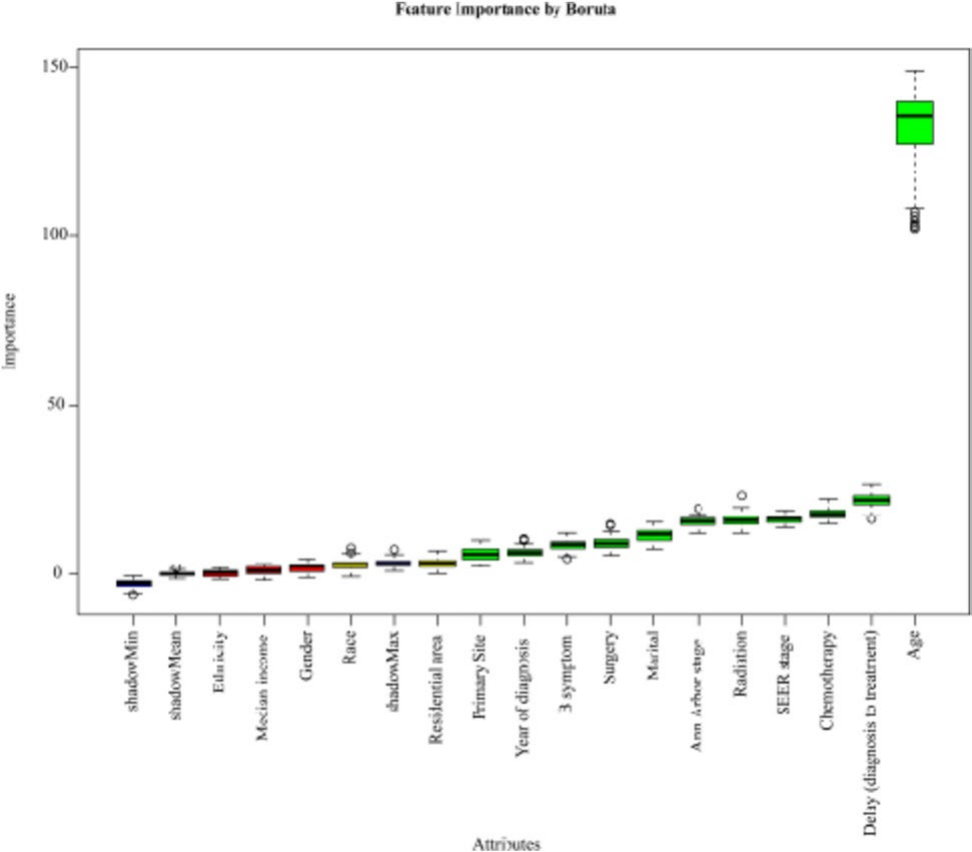
Feature Importance by Boruta Green boxplots represent features with high importance that passed statistical significance testing. Red boxplots indicate low-importance features that failed to meet statistical significance. Blue boxplots represent shadow features.

Feature selection was performed using the Boruta algorithm, and the results were illustrated in Fig. 2. It was observed that primary site, year of diagnosis, B symptoms, surgery, marital status, Ann Arbor stage, radiation, SEER stage, chemotherapy, delay (diagnosis to treatment), age were identified as factors associated with HL OS.

### Predictive performance of the four ML models for OS

Four ML algorithms (Coxph, LightGBM, RSF, and XGBoost) were used for survival analysis and their performance was evaluated using AUC, DCA and Brier score. ALL four ML models showed good prediction performance. In the training set, the RSF model performs best in predicting 1-year OS, 3-year OS, and 5-years OS, with corresponding AUC values of 0.9093 (0.9002, 0.9185), 0.8850 (0.8755, 0.8944), and 0.8778 (0.8685, 0.8870) respectively (Supplement Fig. 1 and Table 2). In the validation set, LightGBM shows the best prediction performance in predicting 1-year OS and 3-year OS, with AUC values of 0.8443 (0.8216, 0.8670) and 0.8128 (0.8006, 0.8429) respectively; when predicting 5-year OS, XGBoost shows the best prediction performance with an AUC value of 0.8257 (0.8065, 0.8449). In the training set, the AUC values of the four ML models decreased over time, while in the validation set, the AUC values first increased and then decreased (Supplement Fig. 2). The results of the DCA showed that for 1-year OS prediction, the net benefit of XGBoost, Coxph, and RSF was significantly higher than that of LightGBM, the treat-all model, and the treat-none model when the threshold probability ranged from 10 to 50%. Similar results were observed for predicting 3-year OS (threshold probability: 10%-62.5%) and 5-year OS (threshold probability: 10%-70%). Similar results were observed in the validation set (Supplement Fig. 3). Table 3 showed the results of the Brier score. For 1-year, 3-year, and 5-year OS, the RSF model had the lowest Brier scores in the training cohort was the lowest, which were 0.0511, 0.0784 and 0.0922, respectively. In the validation cohort, the Brier score of the XGBoost was the lowest in 1-year OS and 5-year OS, which were 0.0537, and 0.1009 respectively; while the Coxph was the lowest in 3-year OS with values of 0.0861. In terms of the trend, the Brier scores of all models increased over time (Supplement Fig. 4). Based on comprehensive comparisons of AUC, DCA, and Brier score, the XGBoost model demonstrated the optimal overall performance.

**Table 2:** Time-Dependent area under the ROC Curve of machine learning-based prognostic models

Model	Times(years)	Training cohort		Validation cohort	
		AUC	95%CI	AUC	95%CI
Coxph	1	0.8450	0.8295–0.8606	0.8428	0.8196–0.8660
	3	0.8195	0.8052–0.8338	0.8212	0.7999–0.8426
	5	0.8125	0.7990–0.8259	0.8244	0.8052–0.8437
LightGBM	1	0.8517	0.8367–0.8668	0.8443	0.8216–0.8670
	3	0.8276	0.8138–0.8414	0.8218	0.8006–0.8429
	5	0.8203	0.8073–0.8334	0.8256	0.8065–0.8447
RSF	1	0.9093	0.9002–0.9185	0.8360	0.8119–0.8602
	3	0.8850	0.8756–0.8944	0.8126	0.7904–0.8347
	5	0.8778	0.8685–0.8870	0.8182	0.7982–0.8381
XGBoost	1	0.8567	0.8419–0.8716	0.8413	0.8178–0.8647
	3	0.8380	0.8248–0.8511	0.8413	0.7994–0.8421
	5	0.8326	0.8201–0.8450	0.8257	0.8065–0.8449

AUC: area under the curve; CI: confidence interval; Coxph: Cox Proportional Hazards; LightGBM: Light Gradient Boosting Machine; Lasso: Least Absolute Shrinkageand Selection Operator; RSF: Random Survival Forests; XGBoost: eXtreme Gradient Boosting

**Fig. 3:**
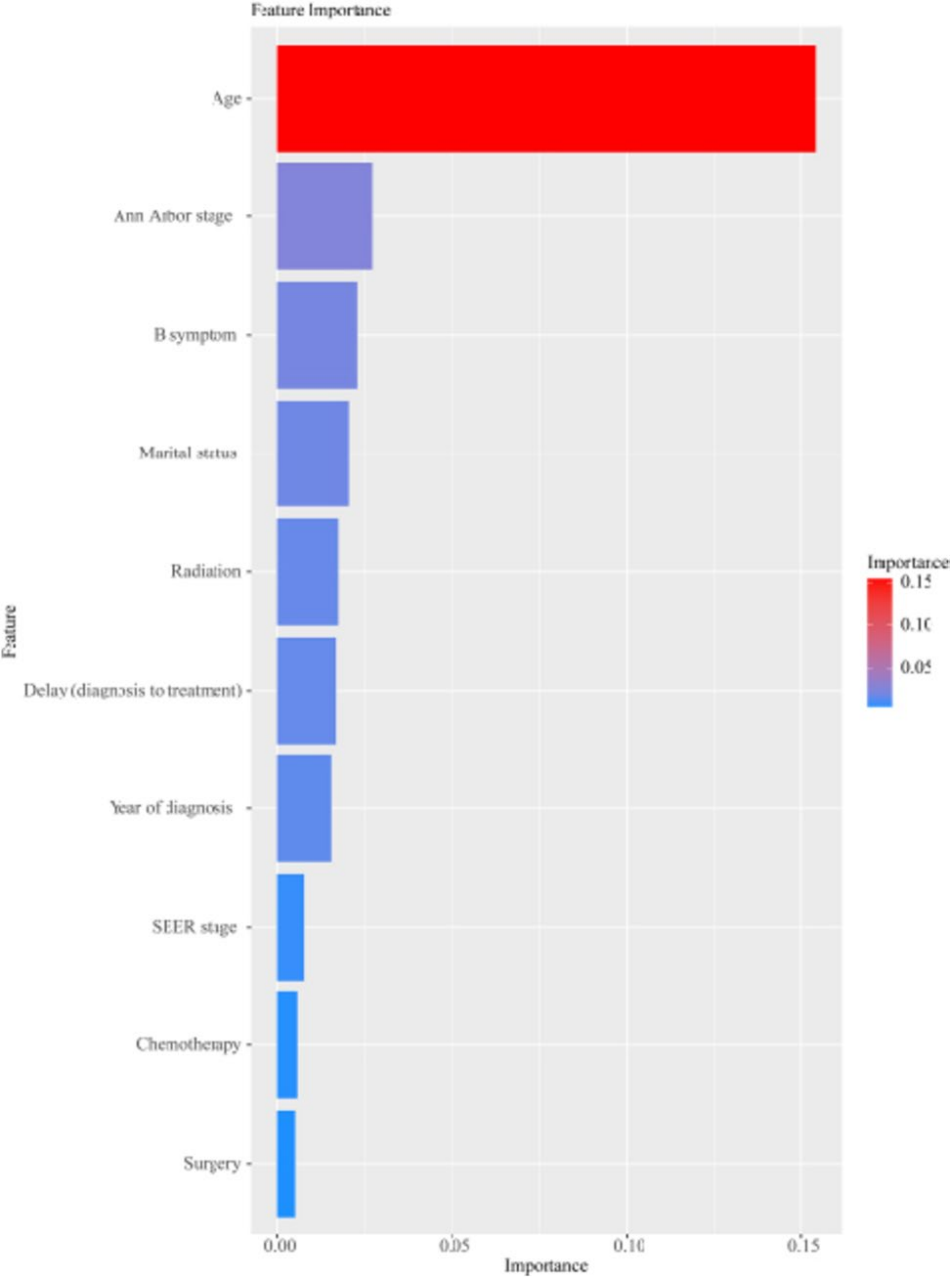
Feature importance ranking. SEER: Surveillance, Epidemiology, and End Results

**Table 3:** Time-Dependent Brier score of machine learning-based prognostic model

Model	Times(years)	Training cohort		Validation cohort	
		Brier	95%CI	Brier	95%CI
Coxph	1	0.0565	0.0526–0.0604	0.0537	0.0478–0.0596
	3	0.0871	0.0826–0.0915	0.0861	0.0792–0.0930
	5	0.1032	0.0986–0.1079	0.1010	0.0938–0.1081
LightGBM	1	0.0684	0.0629–0.0739	0.0637	0.0556–0.0718
	3	0.1145	0.1077–0.1214	0.1135	0.1031–0.1239
	5	0.1425	0.1351–0.1500	0.1421	0.1307–0.1535
RSF	1	0.0511	0.0476–0.0546	0.0538	0.0480–0.0597
	3	0.0784	0.0745–0.0824	0.0870	0.0802–0.0937
	5	0.0922	0.0882–0.0962	0.1016	0.0947–0.1086
XGBoost	1	0.0550	0.0512–0.0589	0.0537	0.0477–0.0596
	3	0.0840	0.0797–0.0883	0.0862	0.0794–0.0930
	5	0.0989	0.0944–0.1034	0.1009	0.0938–0.1080

CI: confidence interval; Coxph: Cox Proportional Hazards; LightGBM: Light Gradient Boosting Machine; Lasso: Least Absolute Shrinkageand Selection Operator; RSF: Random Survival Forests; XGBoost: eXtreme Gradient Boosting

**Fig. 4:**
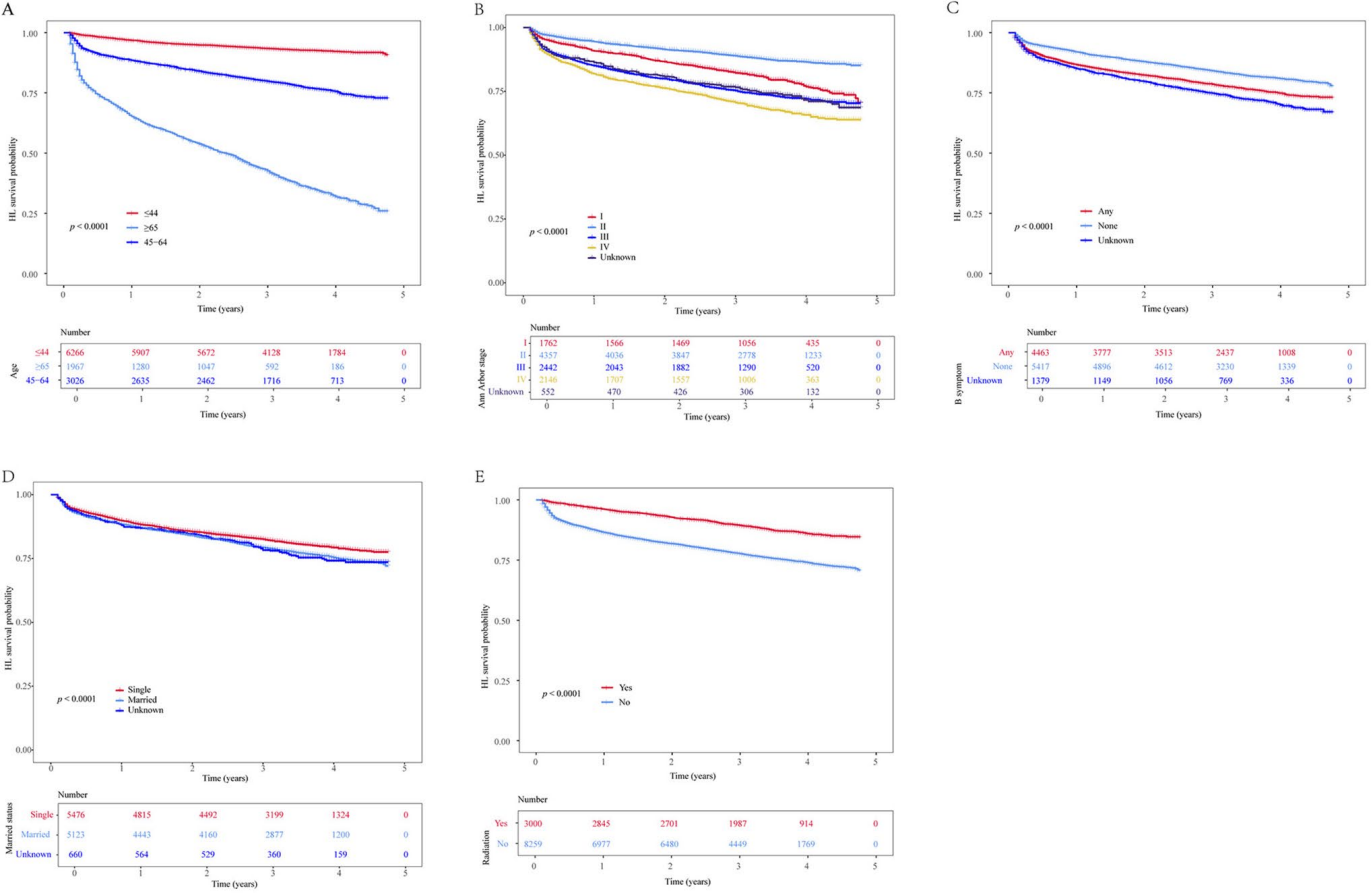
Kaplan–Meier survival analysis (A) Kaplan–Meier survival analysis in different age groups; (B) Kaplan–Meier survival analysis in different Ann Arbor stage; (C) Kaplan–Meier survival analysis indifferent B symptoms; (D) Kaplan–Meier survival analysis in different marital status; (E) Kaplan–Meier survival analysis in with or without radiation treatment. HL: Hodgkin lymphoma

### Feature importance in the ML models

The XGBoost regression, which showed superior performance, was selected for the subsequent SHAP importance analysis. As shown in Fig. 3, age, Ann Arbor stage, B symptoms, marital status, and radiation were the top five indicators in the feature importance ranking for HL OS.

### Survival analysis

Based on the results of the SHAP plot (Fig. 3), the top five indicators in terms of feature importance were selected to compare survival probabilities among different groups. Compared with young patients, those aged 65 years and above had poorer survival outcomes (*P* < 0.001) (Fig. 4 A). Patients with any B symptoms had lower survival probabilities than those none B symptoms (Fig. 4 C). Radiation therapy was associated with a significant survival benefit (*P* < 0.001) (Fig. 4 E). Compared with patients with unknown marital status, single patients had higher survival probabilities (Fig. 4 D). Patients with Ann Arbor stage IV disease had a higher mortality rate than those with lower Ann Arbor stages (Fig. 4 B).

## Discussion

HL is a rare subtype of lymphoma, and with advancements in treatment, its cure rate has exceeded 80%. Although the IPS-7 is useful for evaluating HL prognosis, it is not comprehensive, as it overlooks some important factors. This study collected multiple factors and performed ML models to improve the predictive ability for OS. The key findings were as follows: (1) primary site, year of diagnosis, B symptoms, surgery, marital status, Ann Arbor stage, radiation, SEER stage, chemotherapy, delay (from diagnosis to treatment), and age were associated with OS in HL patients; (2) among the four ML models, XGBoost showed the best comprehensive performance; (3) age, Ann Arbor stage, B symptoms, marital status, and radiation were the most important influencing factors for HL OS.

ML is widely used in cancer research, and HL is no exception. American scholars used convolutional neural networks (CNNs) to classify cell populations affecting classical HL, and found that the AUC of this ML algorithm was 0.92 and the accuracy was 0.824 [35]. Kuang et al. developed ML models to construct a diagnostic model for classical HL with high specificity and sensitivity (AUC = 0.981) [36]. ML models have shown significant superiority in prognostic prediction, with their predictive performance outperforming traditional scoring systems and other conventional methods. A European population-based study compared the predictive performance of ML models, the 3-factor International Prognostic Score (IPS-3), and IPS-7 for OS and progression-free survival (PFS) in advanced HL, finding that ML models had a C-index of 0.789 for OS prediction versus 0.608 (IPS-3) and 0.650 (IPS-7). ML models outperformed IPS-3 and IPS-7 in predicting OS and PFS across both training and validation datasets [37]. Wang et al. applied the Ensemble Algorithm for Cancer Data Clustering to develop a lymphoma prognostic system integrating other prognostic factors, and found that the accuracy of the prognostic system was significantly higher than that of the Ann Arbor system (C-index: 0.6058 vs. 0.6207) [38].

This study found that compared to younger HL patients, elderly HL patients had a higher probability of death, which was associated with increased comorbidities and reduced tolerance to full-course chemotherapy [18]. It is worth noting that there are currently some treatment regimens more favorable to elderly patients, which are effective with few side effects. For example, the MVD regimen, utilizing non-pegylated liposomal doxorubicin without bleomycin and administrable with or without radiotherapy, achieves complete remission in 90% of patients, with an OS rate of 87.5% at 5-year follow-up [39]. In addition, HL patients can benefit from radiation. Radiation is one of the cornerstone treatment modalities for HL, particularly in early-stage patients, where it is often combined with chemotherapy to improve therapeutic efficacy [7]. A multicenter study from India found that among early-stage HL patients, those who received 4 weeks of chemotherapy combined with radiation had a higher 5-year event-free survival (EFS) rate (88.57%) compared to those who received 4 weeks of chemotherapy alone (66.33%) (*P* = 0.0042) [40]. In another single-center study, it was found that patients who received ABVD chemotherapy combined with consolidative radiation had a higher 10-year relapse-free survival rate (100% vs. 47.4%, *P* < 0.001) compared with those who received ABVD chemotherapy alone [41]. Relapsed, refractory, and advanced HL patients can also benefit from external beam radiation, with those receiving radiation achieving prolonged complete remission [42]. In addition, radiation can also reduce the risk of HL recurrence. A phase III study involving 1,150 newly diagnosed early-stage HL patients aged 18–75 found that omitting consolidative radiation resulted in higher rates of local and early relapse [43]. Another study found that short-course radiation can reduce the recurrence of HL [44]. Although existing treatments have improved the HL patient survival, treatment-related toxicity, particularly that of radiation and chemotherapy, cannot be ignored. For example, hematological toxicity is a common adverse event in HL patients receiving anthracycline-based regimens [45]. To mitigate this, aggressive primary prophylaxis against infections is recommended, including the administration of long-acting granulocyte colony-stimulating factor, trimethoprim-sulfamethoxazole, and acyclovir [46, 47]. Therefore, reducing treatment-related toxicity is particularly important.

Although this study is population-based, it has inherent limitations. First, the SEER database lacks detailed treatment information (e.g., drug dosages) and laboratory data (e.g., red blood cell distribution width), and the IPS-7 is not available—all of which are crucial for predicting HL prognosis. Additionally, the study population is from the United States, so the findings may not be generalizable to populations from other countries. Meanwhile, due to the absence of data extracted from other databases, the model was only internally validated rather than externally validated. In the future, more cases and comprehensive patient data should be collected to conduct more in-depth analyses.

## Conclusion

Primary site, year of diagnosis, B symptoms, surgery, marital status, Ann Arbor stage, radiation, SEER stage, chemotherapy, delay (diagnosis to treatment), age were associated with HL OS. Age, Ann Arbor stage, B symptoms, marital status, and radiation were the top five indicators in the feature importance ranking for HL OS. The XGBoost model had excellent predictive performance in HL OS, which further helps clinicians to select appropriate treatment options.

## Supplementary Information

Below is the link to the electronic supplementary material.

Supplementary file1 (PDF 683 KB)

Supplementary file2 (PDF 108 KB)

Supplementary file3 (PDF 173 KB)

Supplementary file4 (PDF 106 KB)

## Data Availability

The datasets are available in the SEER database, http://seer.cancer.gov.
