# Supplementary figures and images for: Predicting survival of Hodgkin lymphoma using machine learning-an analysis based on the SEER database
Source: Ann Hematol. 2026 Apr 30;105(5):277. doi: 10.1007/s00277-026-06791-x (PMC13128760; doi:10.1007/s00277-026-06791-x)

A

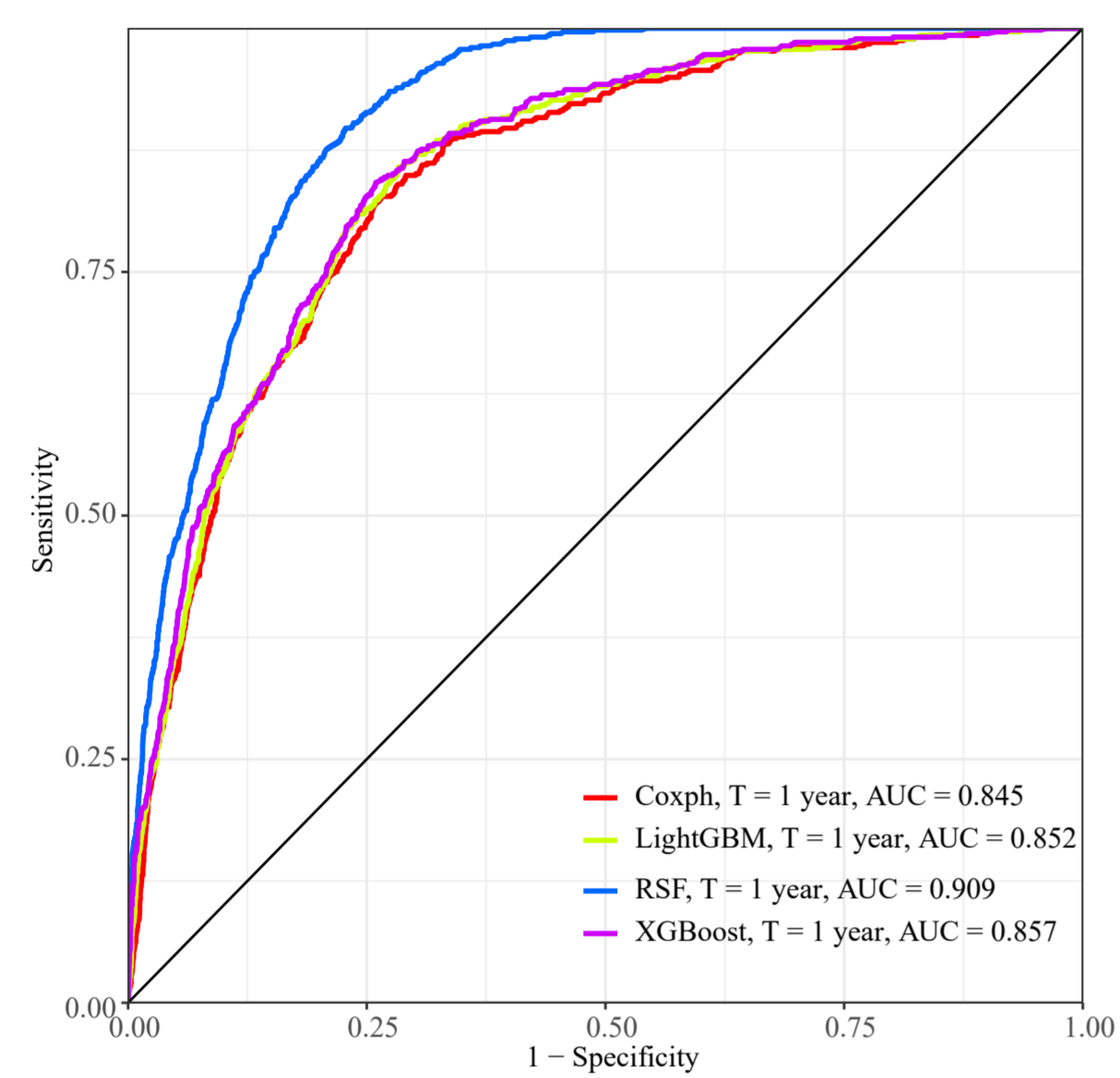

B

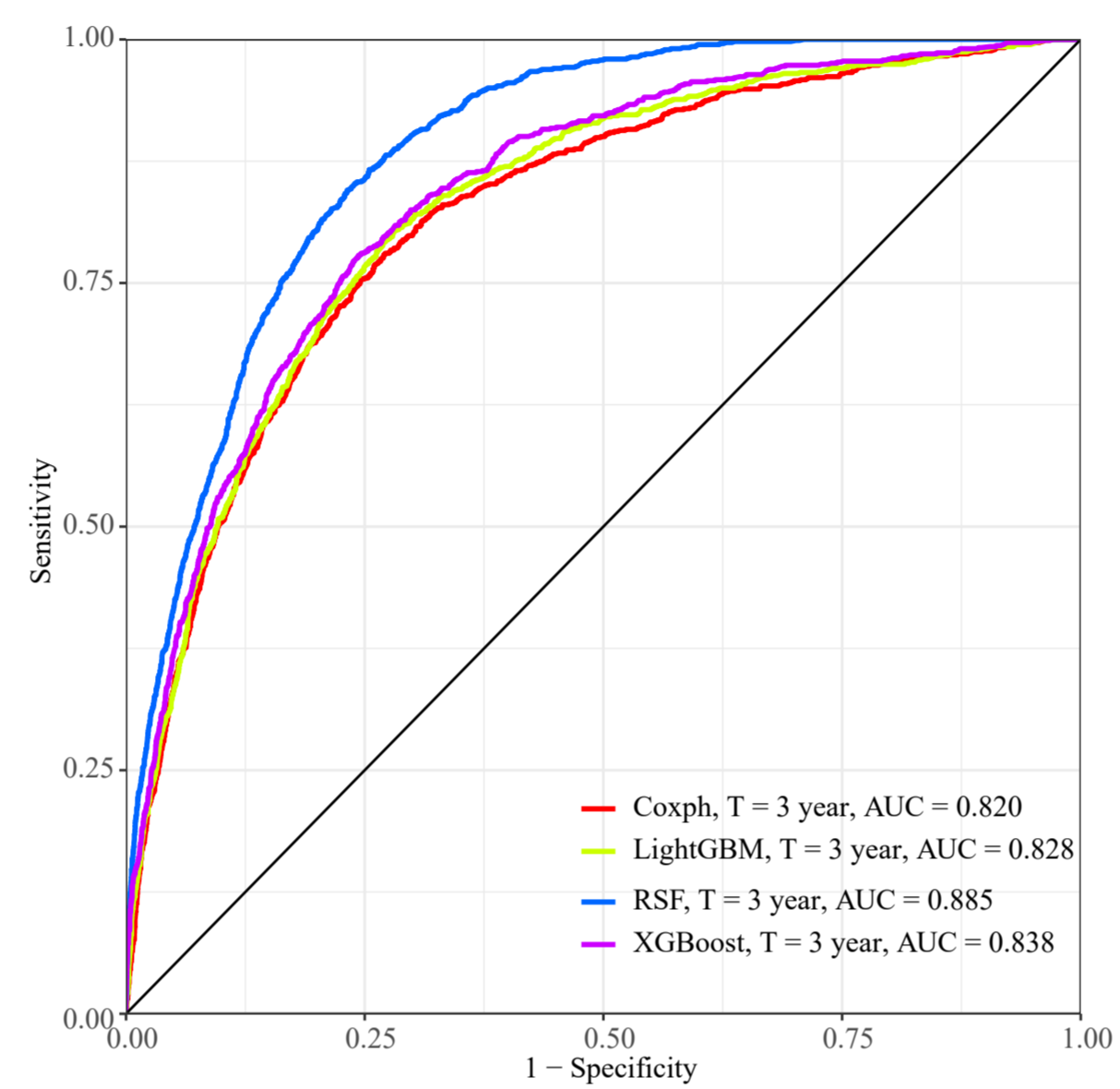

C

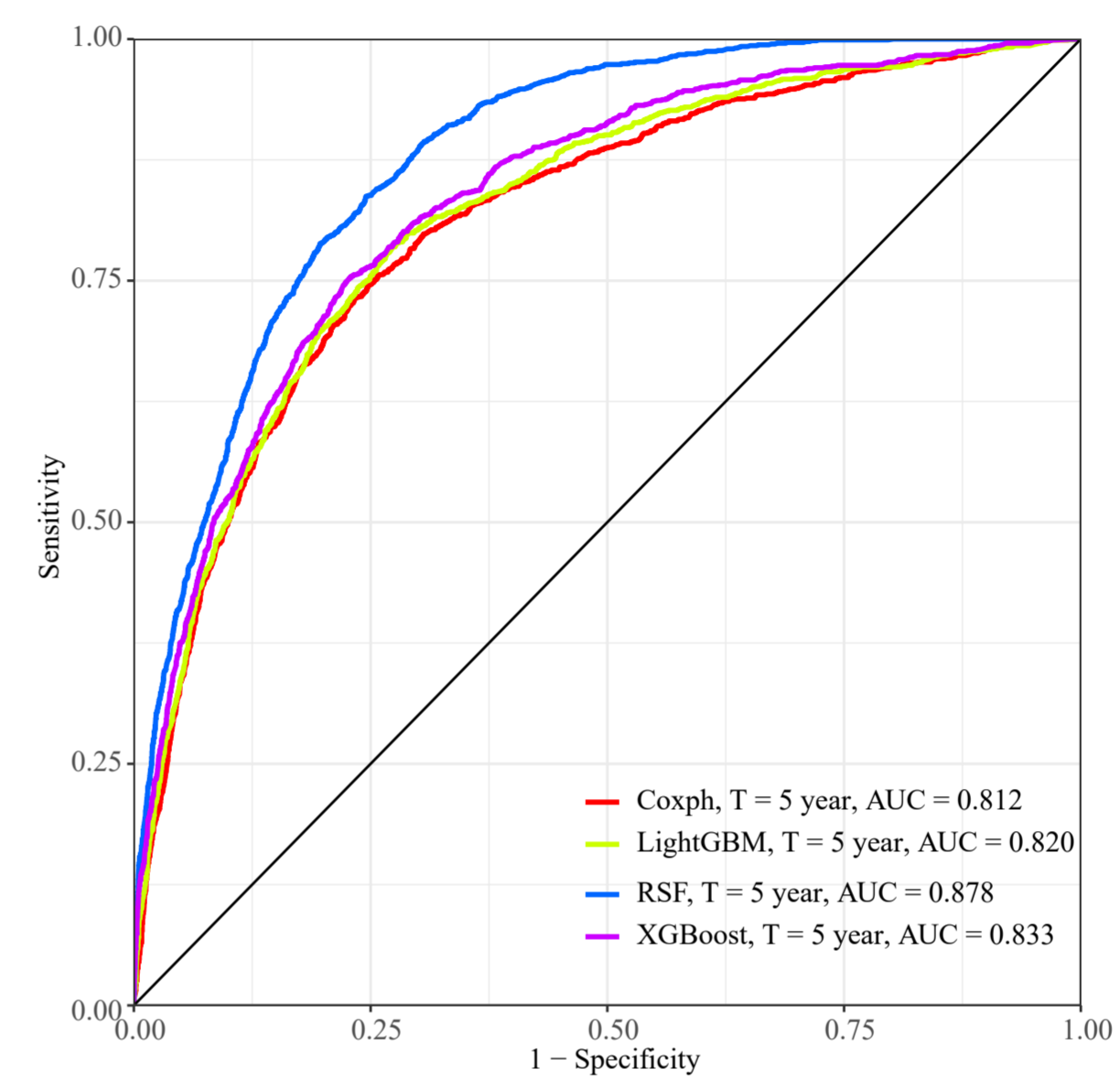

D

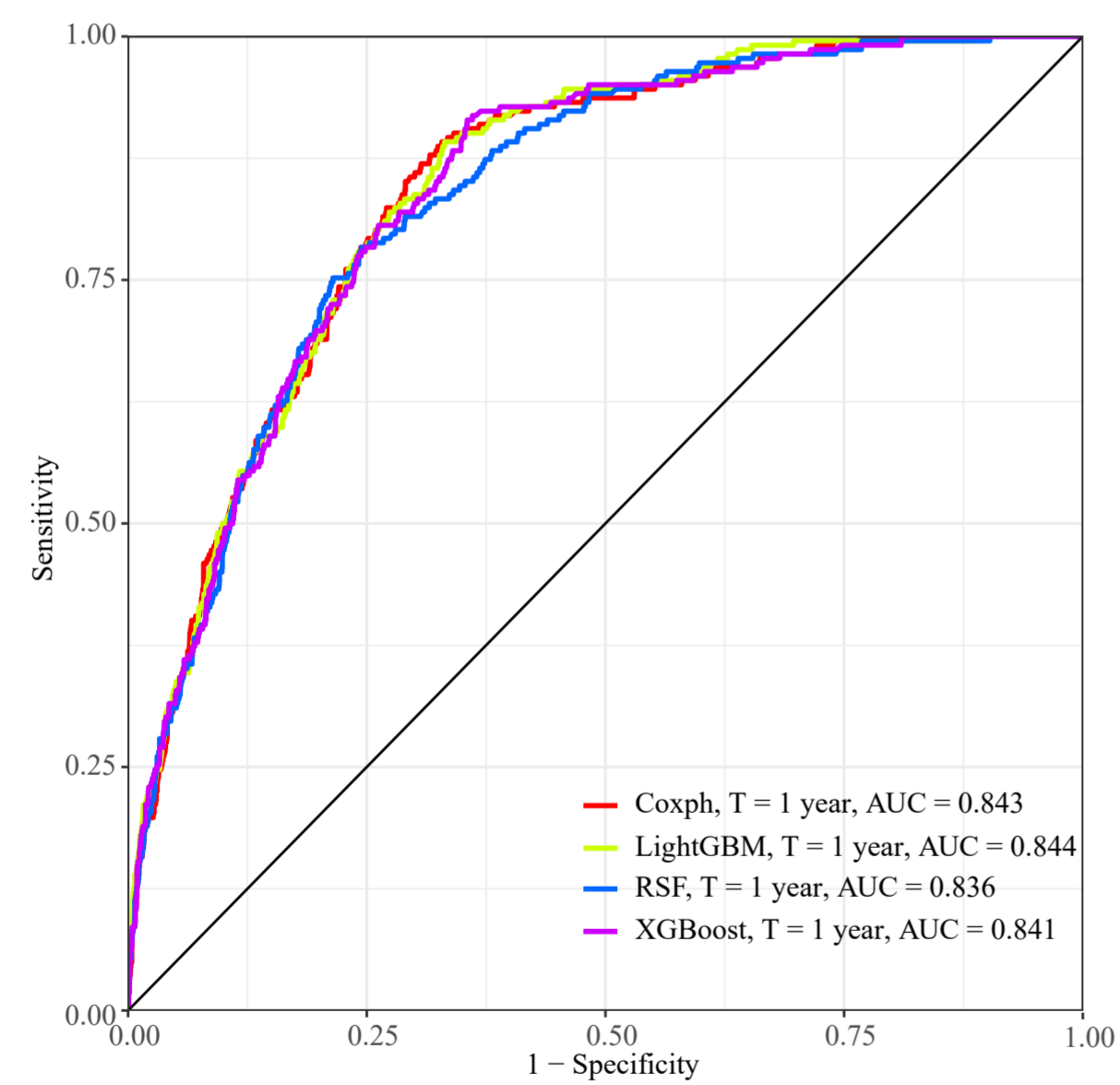

E

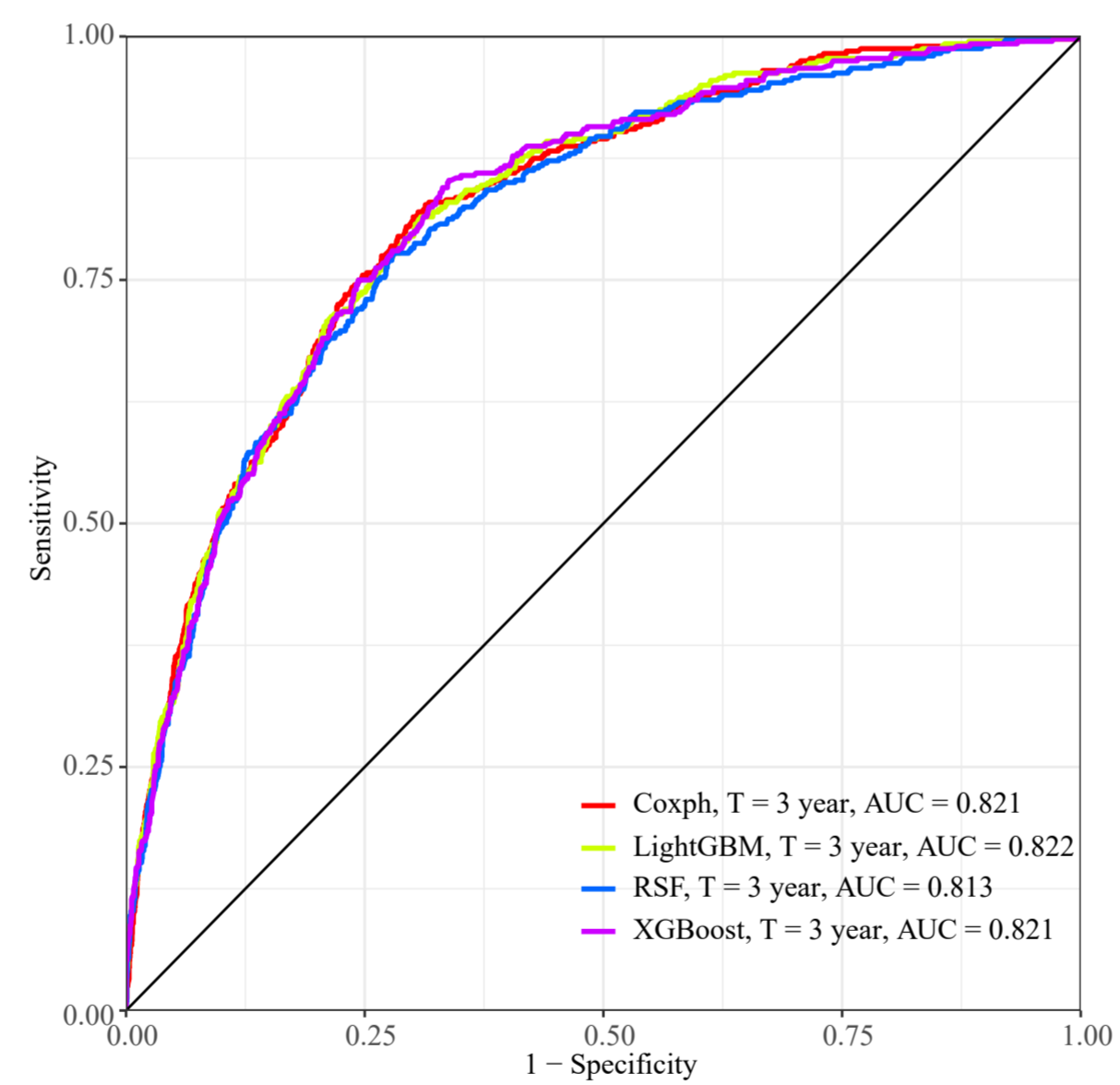

F

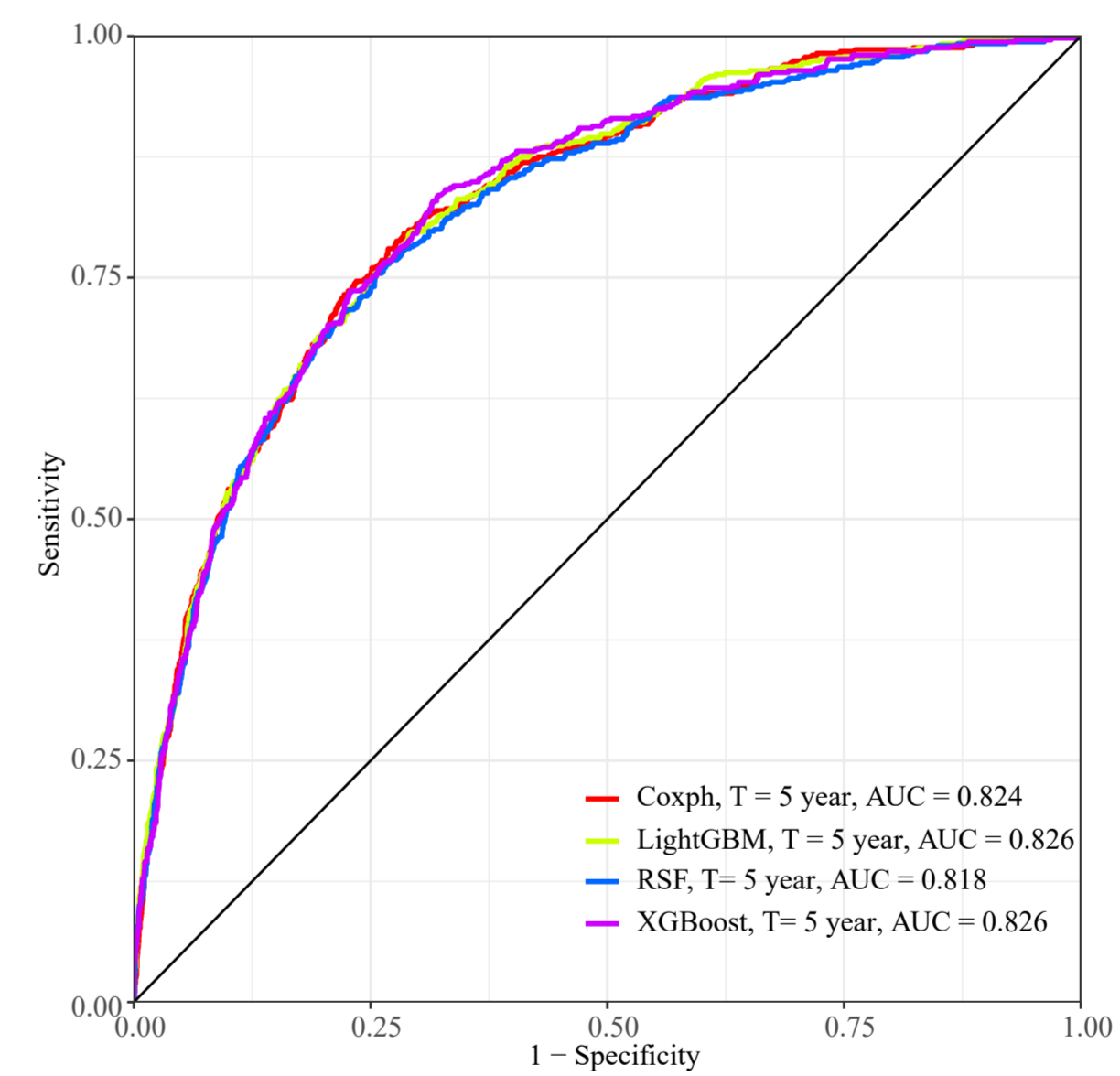

Supplement: Supplementary file 1 — Supplementary file1 (PDF 683 KB) [file 277_2026_6791_MOESM1_ESM.pdf]

A

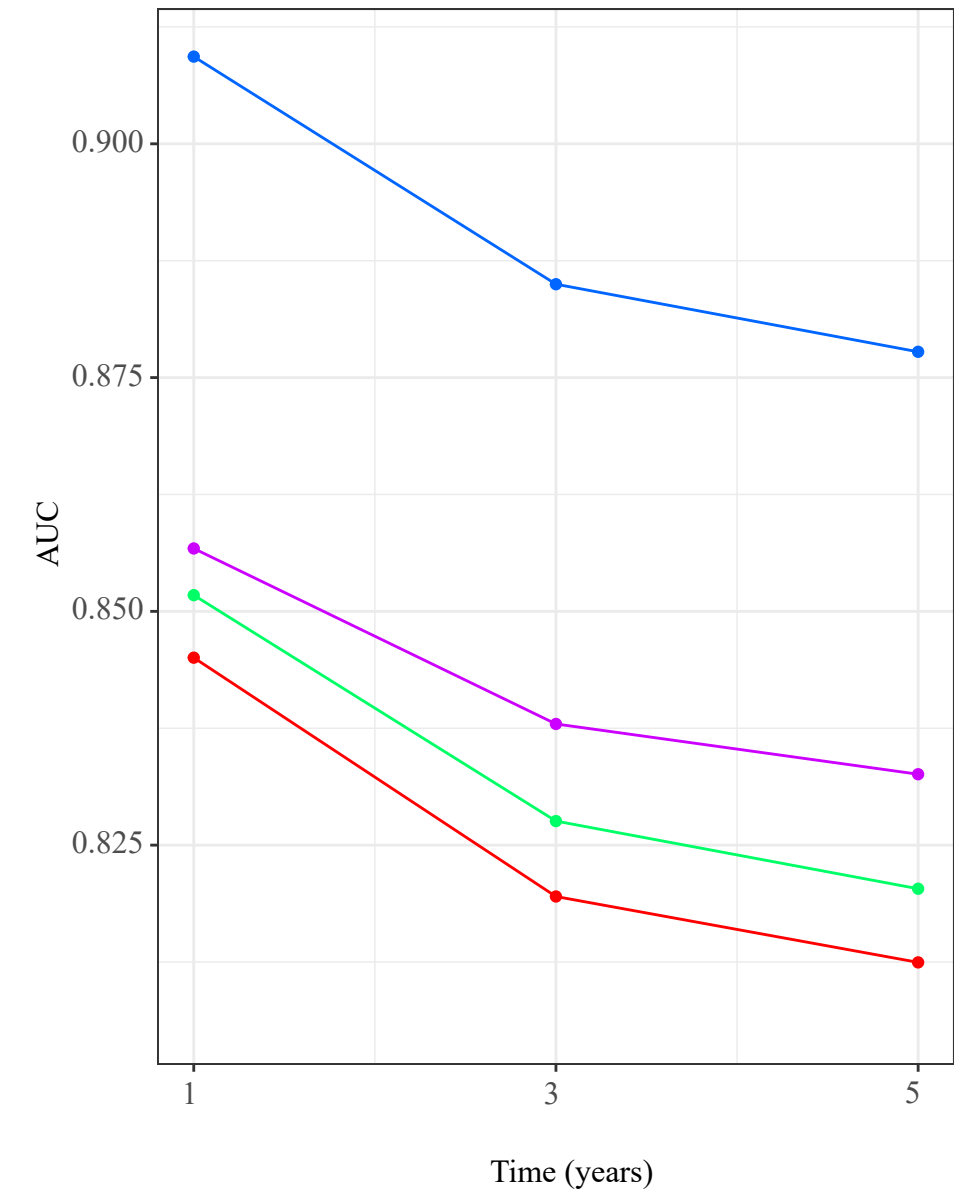

B

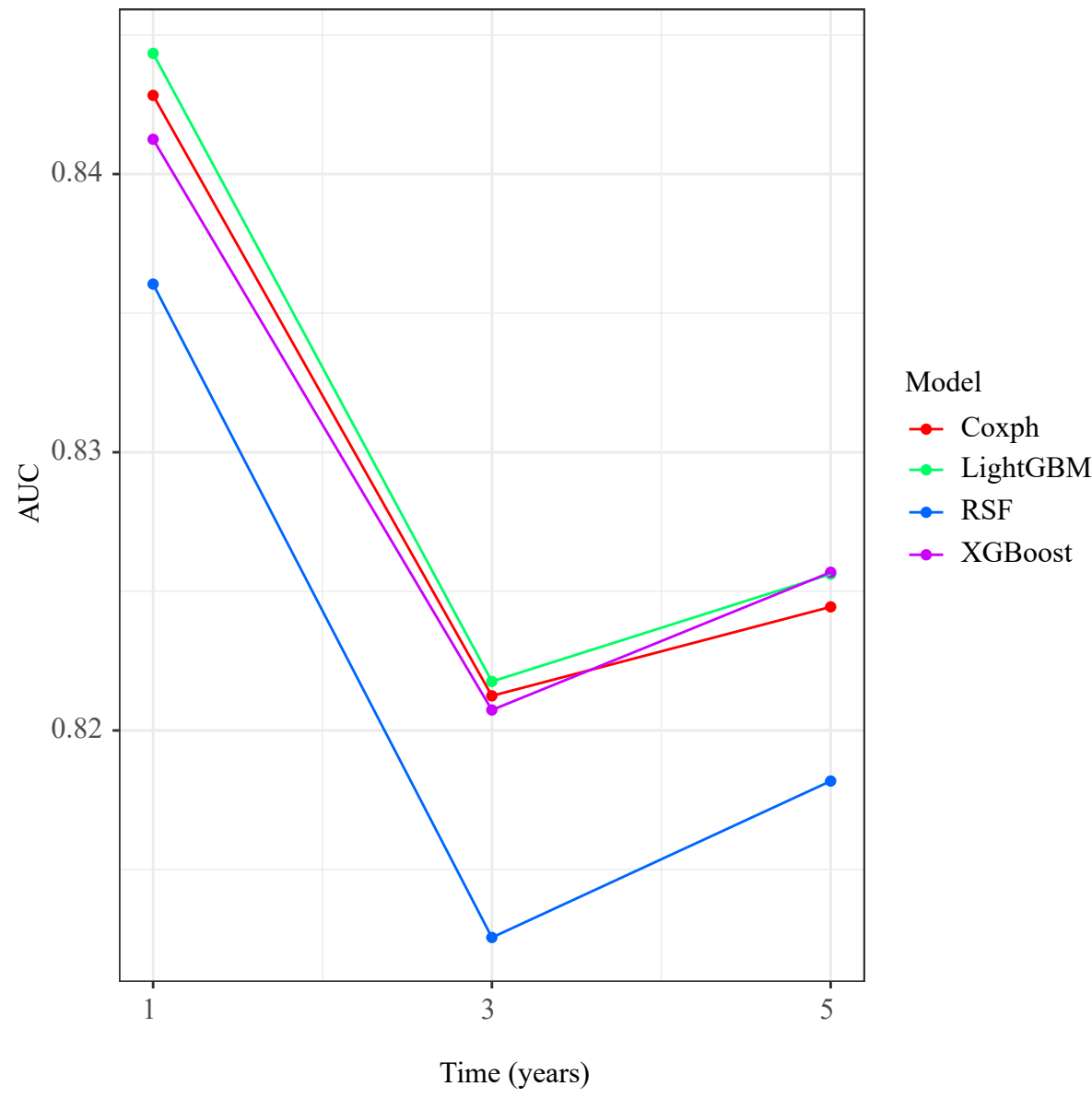

Supplement: Supplementary file 2 — Supplementary file2 (PDF 108 KB) [file 277_2026_6791_MOESM2_ESM.pdf]

A

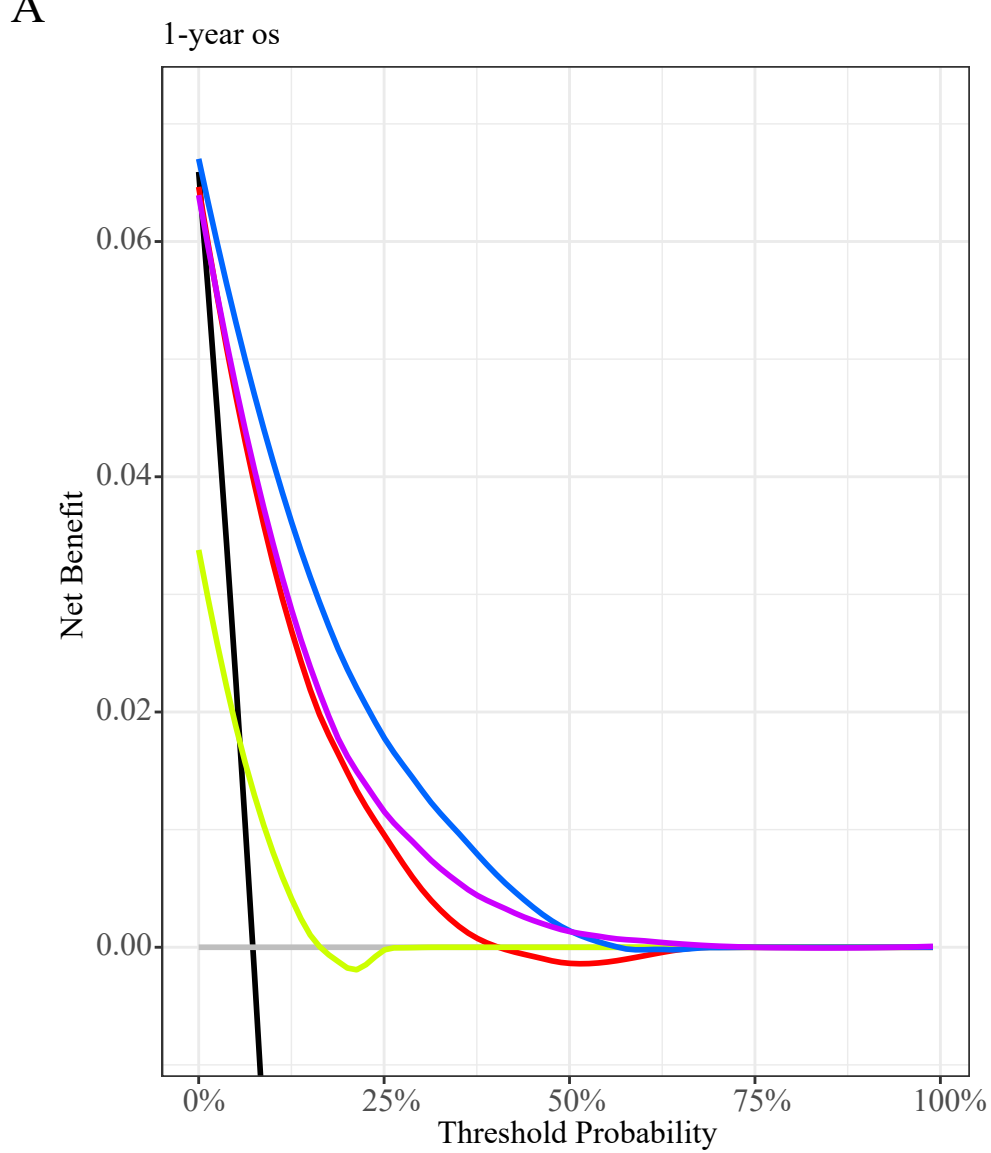

B

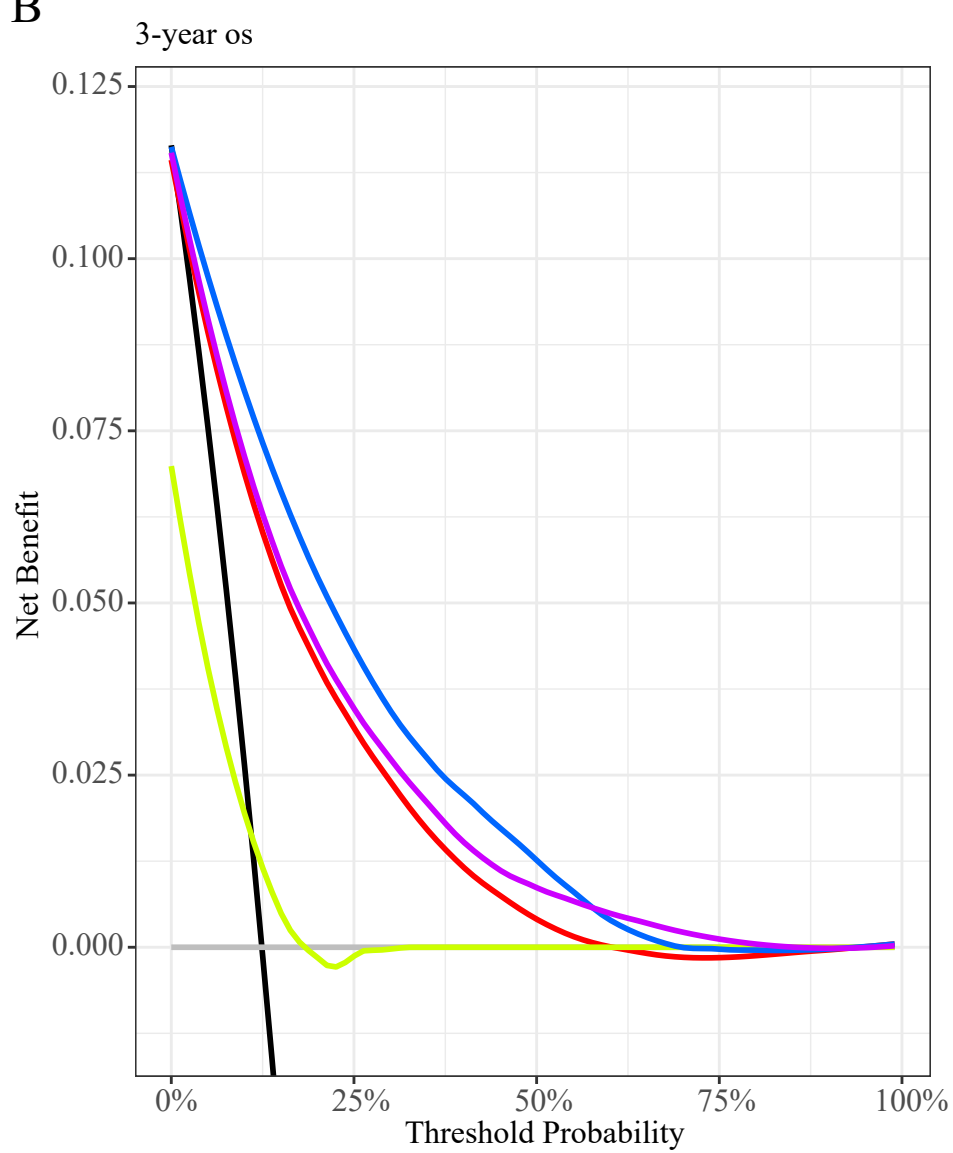

C

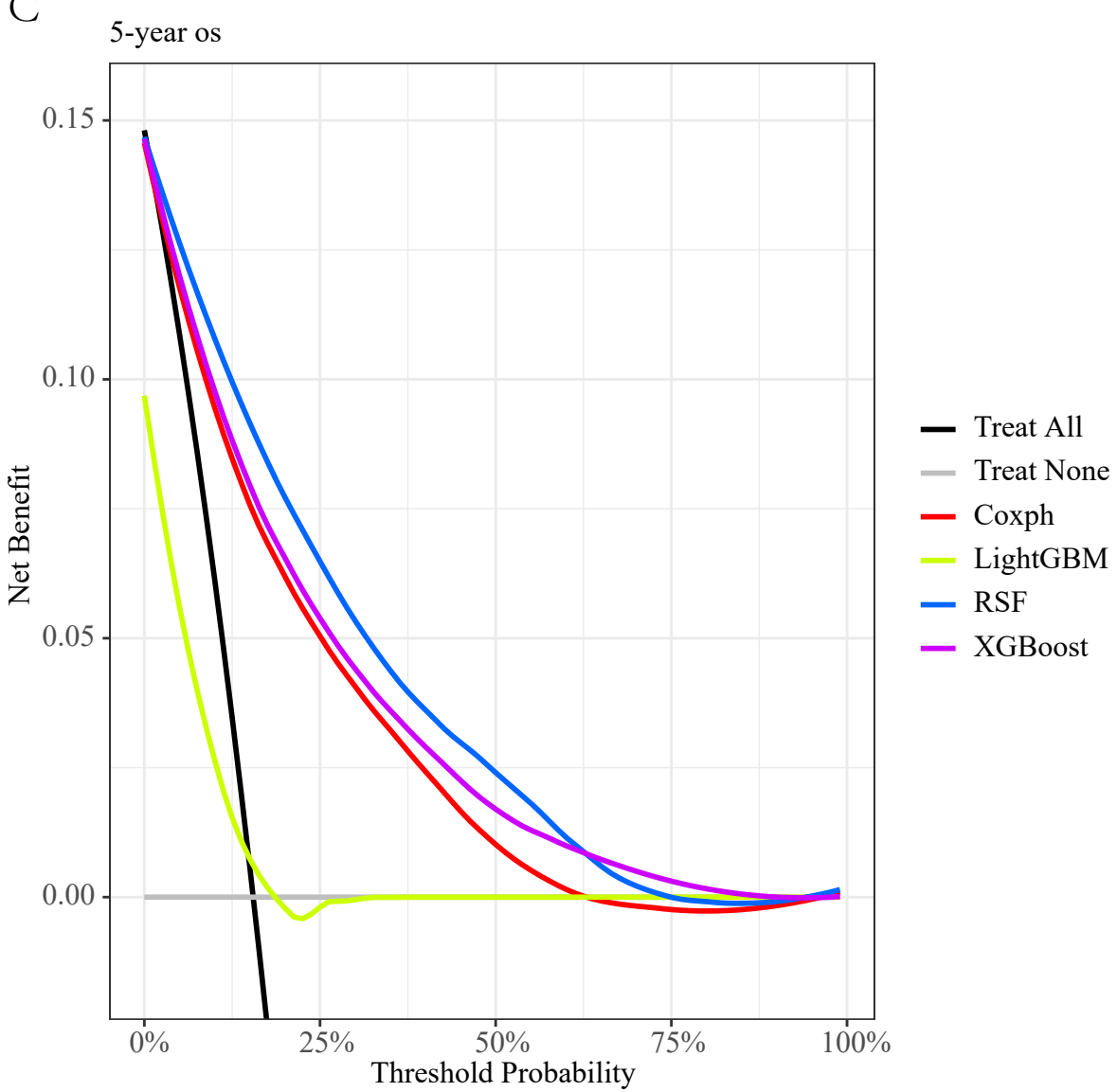

D

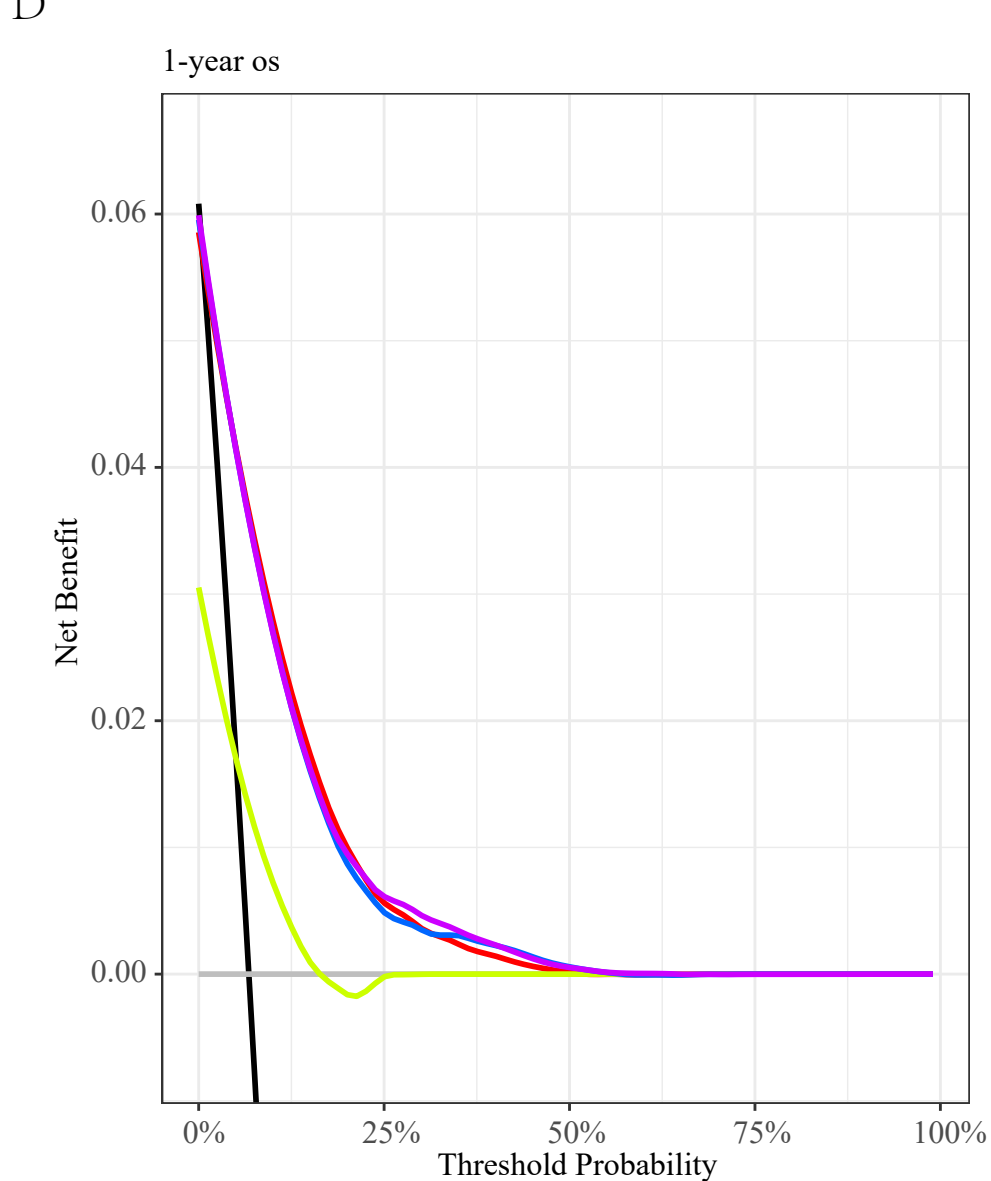

E

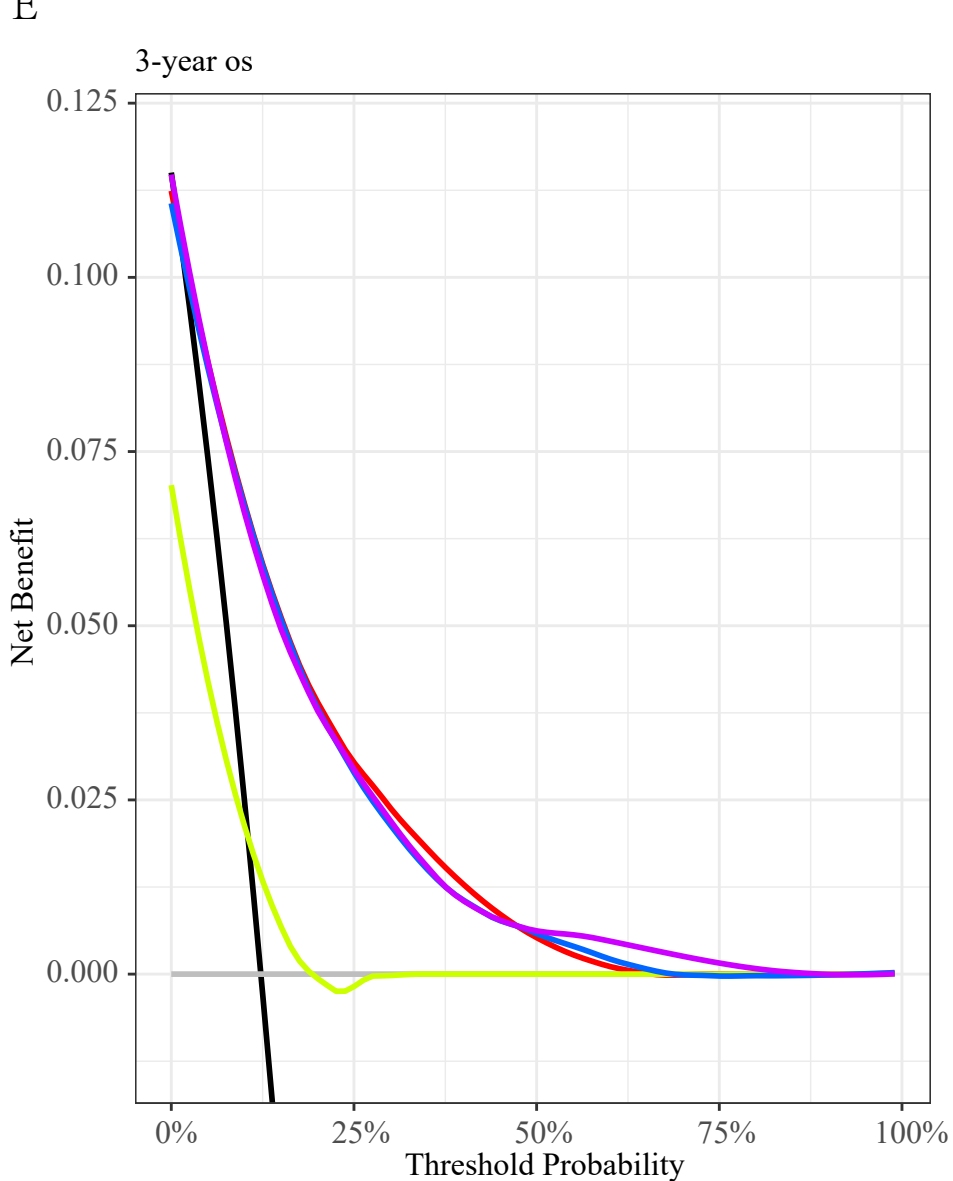

F

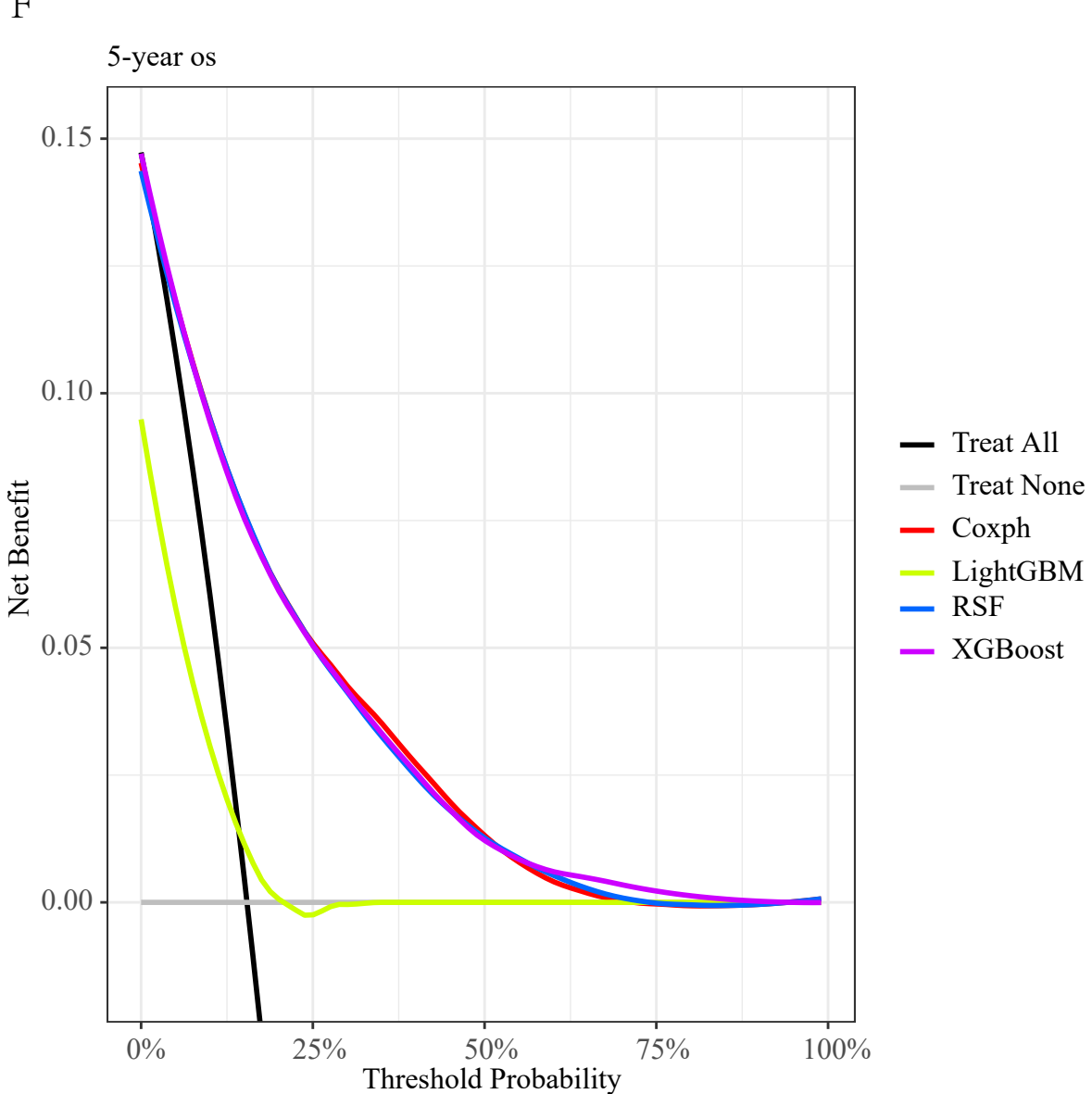

Supplement: Supplementary file 3 — Supplementary file3 (PDF 173 KB) [file 277_2026_6791_MOESM3_ESM.pdf]

A

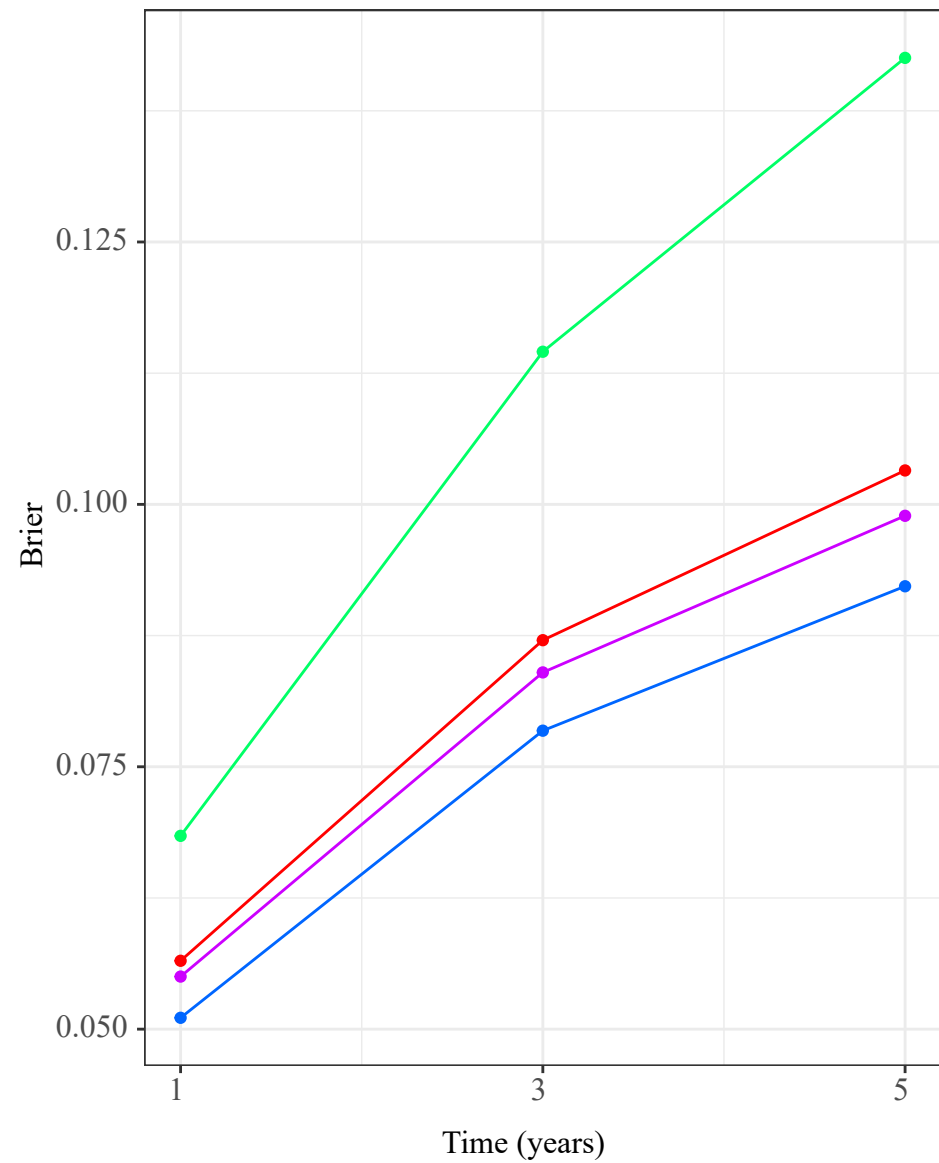

B

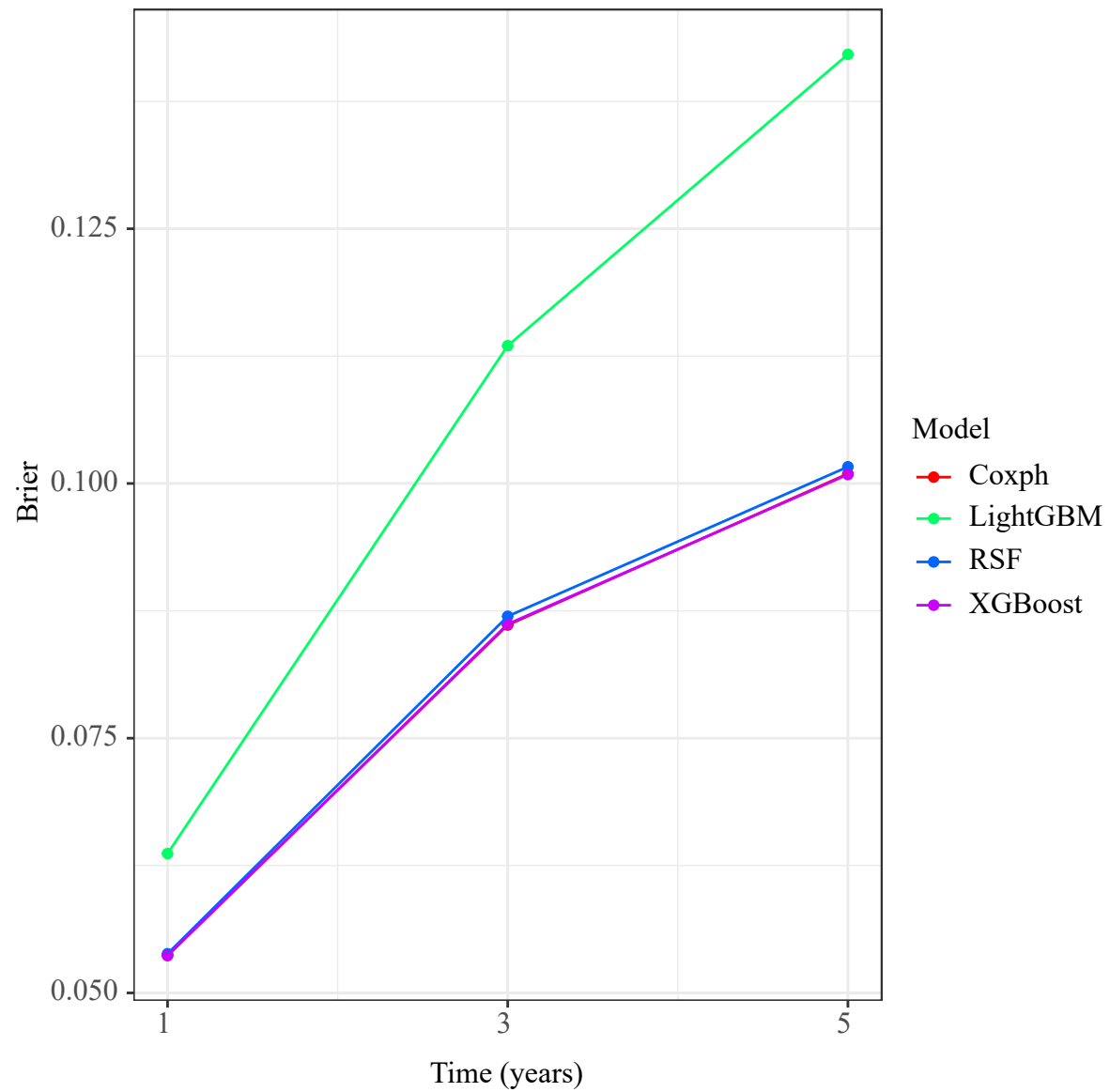

Supplement: Supplementary file 4 — Supplementary file4 (PDF 106 KB) [file 277_2026_6791_MOESM4_ESM.pdf]
